# Therapeutic role of interferon-γ in experimental autoimmune encephalomyelitis is mediated through a tolerogenic subset of splenic CD11b^+^ myeloid cells

**DOI:** 10.1186/s12974-024-03126-3

**Published:** 2024-05-31

**Authors:** Gabriel Arellano, Eric Acuña, Eileah Loda, Lindsay Moore, Juan E. Tichauer, Cristian Castillo, Fabian Vergara, Paula I. Burgos, Pablo Penaloza-MacMaster, Stephen D. Miller, Rodrigo Naves

**Affiliations:** 1 Program of Immunology, Institute of Biomedical Sciences, Faculty of Medicine, Universidad de Chile, Santiago, Chile; 2 Department of Microbiology-Immunology, Feinberg School of Medicine, Northwestern University, Chicago, IL US; 3 Center for Human Immunology, Feinberg School of Medicine, Northwestern University, Chicago, IL US; 4 Department of Clinical Immunology and Rheumatology, School of Medicine, Pontificia Universidad Católica de Chile, Santiago, Chile

**Keywords:** Multiple sclerosis, Experimental autoimmune encephalomyelitis, Interferon-γ, Regulatory T cells, CD11b^+^ cells, TGF-β

## Abstract

Cumulative evidence has established that Interferon (IFN)-γ has both pathogenic and protective roles in Multiple Sclerosis and the animal model, Experimental Autoimmune Encephalomyelitis (EAE). However, the underlying mechanisms to the beneficial effects of IFN-γ are not well understood. In this study, we found that IFN-γ exerts therapeutic effects on chronic, relapsing-remitting, and chronic progressive EAE models. The frequency of regulatory T (Treg) cells in spinal cords from chronic EAE mice treated with IFN-γ was significantly increased with no effect on Th1 and Th17 cells. Consistently, depletion of FOXP3-expressing cells blocked the protective effects of IFN-γ, indicating that the therapeutic effect of IFN-γ depends on the presence of Treg cells. However, IFN-γ did not trigger direct in vitro differentiation of Treg cells. In vivo administration of blocking antibodies against either interleukin (IL)-10, transforming growth factor (TGF)-β or program death (PD)-1, revealed that the protective effects of IFN-γ in EAE were also dependent on TGF-β and PD-1, but not on IL-10, suggesting that IFN-γ might have an indirect role on Treg cells acting through antigen-presenting cells. Indeed, IFN-γ treatment increased the frequency of a subset of splenic CD11b^+^ myeloid cells expressing TGF-β-Latency Associated Peptide (LAP) and program death ligand 1 (PD-L1) in a signal transducer and activator of transcription (STAT)-1-dependent manner. Furthermore, splenic CD11b^+^ cells from EAE mice preconditioned in vitro with IFN-γ and myelin oligodendrocyte glycoprotein (MOG) peptide exhibited a tolerogenic phenotype with the capability to induce conversion of naïve CD4^+^ T cells mediated by secretion of TGF-β. Remarkably, adoptive transfer of splenic CD11b^+^ cells from IFN-γ-treated EAE mice into untreated recipient mice ameliorated clinical symptoms of EAE and limited central nervous system infiltration of mononuclear cells and effector helper T cells. These results reveal a novel cellular and molecular mechanism whereby IFN-γ promotes beneficial effects in EAE by endowing splenic CD11b^+^ myeloid cells with tolerogenic and therapeutic activities.

**Supplementary Information:**

The online version contains supplementary material available at 10.1186/s12974-024-03126-3.

## Introduction

Multiple Sclerosis (MS) is a chronic autoimmune disease of the central nervous system (CNS), characterized by demyelination and axonal damage mediated by myelin-specific T cells [1]. Although the etiology of MS remains unknown, both genetic and environmental factors are involved in breaking self-tolerance [2, 3]. MS is classified into two main clinical forms: relapsing-remitting (RR)-MS, characterized by episodes of acute neuroinflammation followed by variable recovery periods, and progressive MS, consisting of chronic and irreversible neurological disability [4, 5]. Our understanding of the immunopathogenesis of MS and the development of therapies for this disease is largely based on the study of an animal model, experimental autoimmune encephalomyelitis (EAE) [6–9]. EAE is actively induced by immunization with myelin-derived antigens associated with adjuvant and consists of an induction phase and an effector phase. During the inductive phase of EAE, peripheral myeloid antigen-presenting cells (APC) process and present myelin antigens to naïve CD4^+^ T cells inducing differentiation of encephalitogenic interferon (IFN)-γ-producing Th1 and IL-17-producing Th17 cells [10, 11]. Next, during the early effector phase, innate and adaptive immune cells, including Th1 and Th17 cells, migrate from the periphery into the CNS generating a rapid and acute progression of disease leading to chronic demyelination and axonal damage [12]. Therefore, myeloid APC play a critical role in MS and EAE activating encephalitogenic T cells and perpetuating the neuroinflammatory process. However, APC also have the capability to induce activation of Forkhead box p3 (FOXP3^+^) regulatory T (Treg) cells through the secretion of several molecules such as interleukin (IL)-10 and tumor growth factor (TGF)-β, and the interaction of co-inhibitory molecules such as those belonging to the program death-1 (PD-1)/program-death ligands (PD-Ls) system [13–17]. In turn, Treg cells develop a key protective role in EAE and MS, exerting immunosuppressive activities on effector T cells and regulating disease onset and progression [18–23]. Indeed, a functional impairment of Treg cells has been related with MS pathogenesis [24–27].

IFN-γ is an essential cytokine that plays an important role in both innate and adaptive immune response. Its receptor (IFN-γR) is ubiquitously expressed on all nucleated cells in the body [28], primarily signaling through the transcription factor signal transducers and activators of transcription (STAT)-1 [29], to induce expression of multiple interferon-stimulated genes (ISGs) [30, 31]. Historically, IFN-γ is thought to have a pro-inflammatory role in MS and EAE [32–34]. However, several studies have paradoxically shown that IFN-γ can also mediate protective functions in EAE and MS (reviewed in [35, 36]). In MS, induction of endogenous IFN production in progressive-MS patients showed that some patients with improving symptoms had high levels of serum IFN-γ, while worsening clinical symptoms was related to low serum IFN-γ levels [37]. In EAE, cumulative evidence has demonstrated that IFN-γ signaling is necessary to suppress EAE, and that IFN-γ treatment has anti-inflammatory and tolerogenic effects on disease progression [38–40]. Moreover, it has been reported that the therapeutic activity of IFN-β, an extensively used therapy in RRMS patients, depends on IFN-γ signaling because IFN-β treatment has no effect on the development of disease in IFN-γR-deficient mice [41]. Also, we have shown that IFN-γ has a dual role depending on the phase of EAE: playing a pathogenic role during the inductive phase and a protective during the acute effector phase of disease [42]. Similar results have been found using neutralizing antibodies against IFN-γ in EAE mice [43]. Recently, we have reported that IFN-γ administered at the peak of EAE results in amelioration of clinical symptoms and attenuation of neuroinflammation, in a STAT-1-dependent manner. Histological analyses showed that spinal cord sections from IFN-γ-treated EAE mice exhibit significantly less infiltration of inflammatory cells and fewer demyelinated areas compared to control-treated EAE mice. Besides, IFN-γ treatment promoted a shift from activated microglia (MG) to resting MG and the induction of a subset of CX3CR1^high^PD-L1^low^ MG characterized by a homeostatic and anti-inflammatory transcriptional signature [44]. Therefore, accumulative evidence has demonstrated a beneficial role of IFN-γ in EAE and MS. However, after 30 years of study, the mechanisms underlying the protective effects of IFN-γ in these pathologies remain largely unknown. Because EAE and MS are diseases mainly mediated by myelin-specific T cells, in this study we addressed the impact of IFN-γ treatment on peripheral and CNS infiltrating CD4^+^ T cell subpopulations, including Th1, Th17, and Treg cells, in EAE. We found that the therapeutic effect of IFN-γ requires the presence of functional FOXP3^+^ Treg cells, although IFN-γ does not directly regulate Treg cell differentiation. Instead, IFN-γ acts directly on splenic CD11b^+^ cells inducing a tolerogenic phenotype in a STAT-1-dependent manner. Interestingly, IFN-γ-stimulated splenic CD11b^+^ cells induce in vitro conversion of naïve CD4^+^ T cells to Treg cells mediated by secretion of tumor growth factor (TGF)-β. Furthermore, adoptive transfer of splenic CD11b^+^ cells from IFN-γ-treated EAE mice to untreated EAE mice induces disease amelioration by reducing CNS infiltration of effector helper T cells.

## Materials and methods

### Mice

C57BL/6 (CD45.1, CD45.2, and *Stat-1*^*−/−*^) mice, non-obese diabetic (NOD) mice, and SJL/J mice were purchased from The Jackson Laboratory (US). The C57BL/6-FOXP3^GFP − DTR^ (FOXP3-DTR) mice were kindly provided by Dr. Pablo Penaloza-MacMaster (Northwestern University, US). Mice were maintained and bred at the animal core facility of Faculty of Medicine-Universidad de Chile or Center for Comparative Medicine-Northwestern University and treated in accordance with the Institutional Animal Care and Use guidelines (IACUC) of the Universidad de Chile and Northwestern University.

### EAE induction, clinical scoring, and treatments

Mice 8 to 12-weeks-old were induced with different types of EAE. Chronic EAE was induced by subcutaneous (s.c.) immunization of C57BL/6 male mice with 150 µg MOG_33 − 55_ peptide (CPC Scientific, California, US) and 500 µg *Mycobacterium tuberculosis* (MT) H37Ra (BD Difco, Detroit, US) in incomplete Freund’s adjuvant (BD Difco, Detroit, US) followed by an intraperitoneal (i.p.) injection with 200 ng pertussis toxin (PTx) (Calbiochem, Campbell, US) on days 0 and 2 post-immunization (p.i.). RR-EAE was induced by s.c. immunization of SJL female mice with 150 µg PLP_139 − 151_ peptide. Chronic progressive EAE was induced in NOD female mice by s.c. immunization with 150 µg MOG_33 − 55_ peptide, 400 µg MT, and a single i.v. injection of 200 ng PTx on day 0. For treatment with IFN-γ, 1 µg/day of recombinant murine IFN-γ (Biolegend, San Diego, US) was administered i.p. for 5 days in chronic and RR-EAE or for 10 days in progressive EAE. For in vivo neutralization of PD-1, mice received two i.p. doses of 500 µg of anti-PD-1 (clone RMP1-14) or isotype Ig control (rat IgG2a, clone 2 A) at day − 2 and 0 of IFN-γ treatment. For in vivo cytokine neutralization, three doses of 100 µg anti-TGF-β (clone 1D11.16.18) and anti-IL-10 (clone JES5-2A5), or their isotype Ig control (mouse anti-IgG1, clone MOPC-2, or rat anti-IgG1, clone HRPN, respectively), were administered i.p. at days 0, 2, and 4 after IFN-γ treatment. All neutralizing antibodies and isotype controls were purchased to BioXcell (Lebanon, US). To deplete FOXP3^+^ Treg cells, FOXP3-DTR mice were i.p. treated with one dose of 2.5 µg of diphtheria toxin (Sigma-Aldrich, Saint Louis, Missouri, USA) at day − 2 and 0 before starting IFN-γ treatment. EAE symptoms were scored using a 0–5 scale as follows: 0, no symptoms; 1, loss of tail tone; 2. flaccid tail; 3, partial paralysis of lower extremities; 4, complete paralysis of lower extremities; 5, moribund/death (animals are humanely euthanized).

### CD4^+^ T cell and APC phenotyping

Mice were perfused intracardially with PBS and brains and spinal cords were isolated and incubated with DNAse (10 units/mL) (New England Biolabs, Ipswich, US) and collagenase D (0.5 mg/mL) (Roche, Manheim, Germany) for 1 h at 37 °C. Then, tissues were disaggregated and centrifuged. Mononuclear cells were obtained using a discontinuous Percoll gradient (Amersham, Piscataway, US). Draining cervical/axillary lymph nodes (LN) and spleens were screened to yield single cell suspensions and splenocytes were treated with red blood lysis buffer (155mM NH4Cl, 10mM KHCO3, y 1mM EDTA, pH 7.3). For T cell flow cytometry analysis, cells were first stimulated with PMA (50 ng/mL)/ionomycin (500 ng/mL) (Sigma-Aldrich, Saint Louis, US) and Brefeldin A (5 µg/mL) (Sigma-Aldrich, Saint Louis, US) for 4 h. Then, cells were stained with LIVE/DEAD™ Fixable dye-Dead Cell Stain Kit (Thermofisher, Carlsbad, US) to determine viability, blocked with Fc Block (Biolegend, San Diego, US) and fixed/permeabilized with FOXP3/transcription factor staining buffer set (eBioscience, San Diego, US) for intracellular staining. A T-cell staining panel consisting of the following markers was used: CD45, CD3, CD4, CD25, FOXP3, IFN-y and IL-17 A. The APC staining panel included the following markers: CD45, CD11b, TGF-β-Latency Associated Peptide (LAP), PD-L1, PD-L2, CD80, CD86 and MHC-II. All antibodies were purchased from Biolegend (San Diego, California, US). Samples were acquired with FACS LSR Fortessa at University of Chile or FACS Celesta (BD bioscience, New Jersey, US) at Northwestern University and analyzed with Flowjo X v10.7 software (Tree Star, Ashland, Oregon, US). Gating flow cytometry strategies are represented in Supplementary Fig. 1.

### Cell purification

CD4^+^CD25^−^ and CD4^+^CD25^+^ T cells were isolated by immunomagnetic selection using the EasySep™ mouse CD4^+^CD25^+^ regulatory T cell isolation kit II (> 90% purity). CD4^+^CD62L^+^CD44^low^ naïve CD4^+^ T cells were isolated using the EasySep™ mouse CD4^+^ T cell isolation kit (> 95% purity). CD11b^+^ cells were purified using the EasySep™ mouse CD11b positive selection kit II (> 87% purity). All purification kits were from Stem Cell (Vancouver, Canada).

### Treg cell induction by IFN-γ

CD4^+^CD25^−^ and CD4^+^CD25^+^ T cells isolated from the spleen and LN of mice at the peak of the disease (day 12–15 p.i.) were cultured with serum-free X-VIVO 20 medium (Lonza, Basel, Switzerland) in the presence of 2 µg/mL of plate-bound anti-CD3 antibody and 1 µg/mL of soluble anti-CD28 antibody (both from Biolegend, San Diego, California, US), with or without IFN-γ, at 37 °C for 3 days. Different concentrations of IFN-γ (1, 10, 25, 50, and 100 ng/mL) were tested (Supplementary Fig. 3B) to select the final concentration of 25 ng/mL, which was previously described to induce Treg cells in similar conditions [45]. Controls with 5 µg/mL anti-IFN-γ antibody or isotype control antibody were used. Similar experiments were also performed in the presence of TGF-β (2 ng/mL) and IL-2 (10 ng/mL) (both from Thermofisher, Waltham, Massachusetts, US) to induce differentiation of Treg cells (iTreg conditions). Cell culture supernatants were collected and stored frozen for further cytokine analysis. The frequency of FOXP3^+^ Treg cells and the expression of CTLA-4, LAG-3, HELIOS, and TGF-β-LAP were determined by flow cytometry.

### Treg cell induction by IFN-γ-stimulated CD11b^+^ cells

CD11b^+^ cells isolated from the spleen of EAE mice at the peak of the disease (day 12–15 p.i.) were cultured in the presence of 10 µg/mL MOG_33 − 55_ peptide and stimulated with 25 ng/mL IFN-γ for 24–72 h. The expression of TGF-β-LAP, PD-L1, PD-L2, CD80, CD86, and MHC-II was determined by flow cytometry. Cell culture supernatants were collected to determine TGF-β levels. In other assays, CD11b^+^ purified cells from the spleen of EAE mice were preconditioned with 10 µg/mL MOG_33 − 55_ peptide and 25 ng/mL IFN-γ for 24 h, washed, and co-cultured with naïve CD4^+^ T cells (ratio 1:1) from non-immunized mice in the presence of 1 µg/mL soluble anti-CD3 antibody (Biolegend, San Diego, California, US) and with either 5 µg/mL anti-TGF-β antibody (BioXcell, Lebanon, US) or anti-PD-L1 antibody (Thermofisher, Carlsbad, US), and their isotype control antibodies. After 72 h, the frequency of Treg (CD4^+^CD25^+^FOXP3^+^) cells was determined by flow cytometry.

### Adoptive transfer of splenic CD11b^+^ cells from IFN-γ-treated EAE mice into recipient EAE mice

CD11b^+^ cells (1 × 10^6^) were purified from spleens of CD45.1^+^ EAE mice treated with IFN-γ or PBS for 5 days and i.v. transferred into untreated CD45.2^+^ EAE mice at the peak of disease. After 3 days, splenocytes and mononuclear cells from spinal cords of recipient EAE mice were isolated and absolute cell number and frequencies of mononuclear cells, CD45.2^+^ cells, CD45.1^+^CD11b^+^ cells, CD3^+^ T cells, CD3^+^CD4^+^ T cells, CD3^+^CD4^−^ T cells, CD3^+^CD4^+^CD25^+^FOXP3^−^ T cells, and Treg cells (CD3^+^CD4^+^CD25^+^FOXP3^+^) were determined by flow cytometry.

### Cytokine measurements

Cell culture supernatants were used to determine the levels of TGF-β1, IL-10, IL-27, Granzyme B, and sFASL by MILLIPLEX® Multiplex Assay kits (Millipore-Sigma, Saint Louis, US) and quantitated using the Luminex® 200™ System (Northbrook, Illinois, US).

### Statistical analysis

Results were analyzed using a Mann–Whitney *U* test or Kruskal-Wallis test. For comparison of EAE scores between two or multiple groups, we conducted two-way ANOVA or one-way ANOVA followed by multiple comparisons with Bonferroni post-hoc test. Statistical analyses were performed with GraphPad Prism v.9.0 software (San Diego, California, US). All data are presented as mean ± SEM. Values were considered statistically significant when *p* < 0.05.

## Results

### Therapeutic effect of IFN-γ is dependent on FOXP3^+^ Treg cells in EAE

**Fig. 1:**
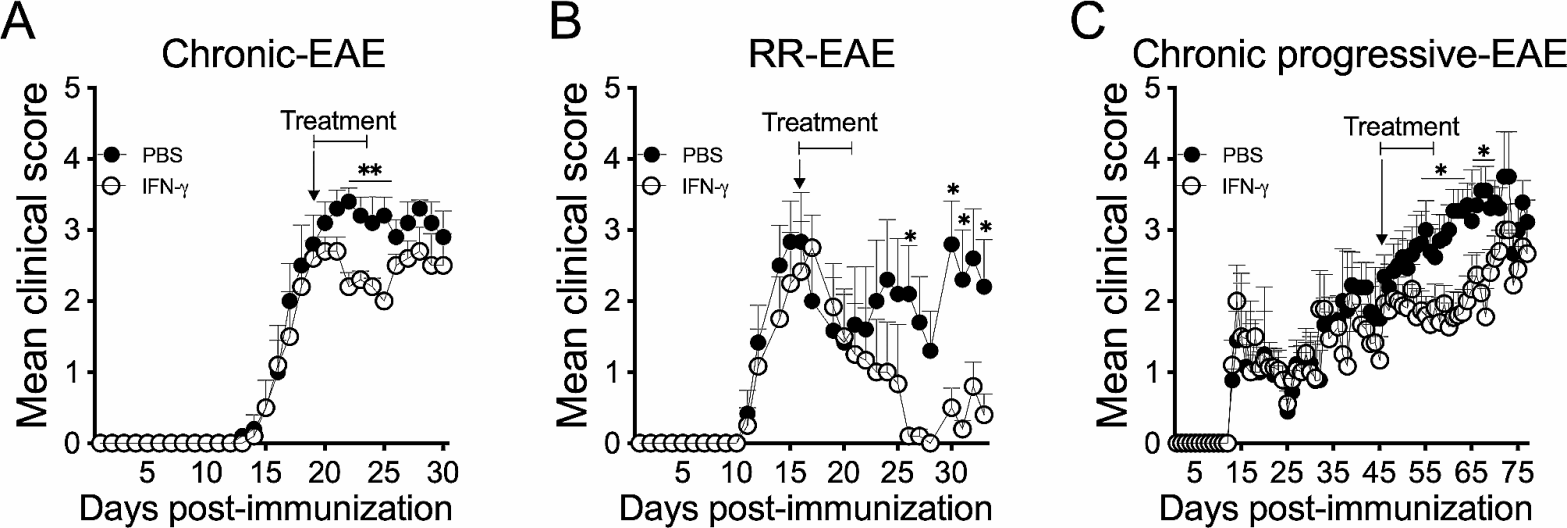
Therapeutic administration of IFN-γ ameliorates clinical severity in chronic, relapsing-remitting, and chronic progressive-EAE. (**A**) Two groups of C57BL/6 mice were induced with chronic EAE (*n* = 9/group) and daily treated with 1 µg IFN-γ (empty black circles) or PBS (solid black circles) at peak of disease for 5 days. (**B**) SJL mice were induced with relapsing-remitting (RR)-EAE (*n* = 6/group) and daily treated with 1 µg IFN-γ (empty black circles) or PBS (solid black circles) at peak of the first flare for 5 days. (**C**) Chronic progressive-EAE was induced in NOD mice (*n* = 13/group) and treated with 1 µg IFN-γ (empty black circles) or PBS (solid black circles) during the chronic phase of the disease (starting at 45 days post-immunization) for 10 days. Clinical symptoms were monitored daily using the standard scoring scale. Results are shown as the mean ± SEM. Data were analyzed using two-way ANOVA followed by Bonferroni post-hoc test. *p < 0.05, **p < 0.01

First, we confirmed that treatment of C57BL/6 mice developing chronic EAE with IFN-γ for 5 days starting at peak of disease induced a significant suppression of clinical symptoms. After cessation of treatment, disease severity slowly returned to levels similar to control EAE mice (Fig. 1A). Next, we asked whether IFN-γ might have beneficial effects in two other clinically distinct EAE models mimicking RRMS (SJL/J mice) and chronic progressive MS (NOD mice). The results showed that IFN-γ administered at the peak of the first flare of RR-EAE (SJL mice) induced a significant attenuation of clinical symptoms and prevention of further relapses compared to PBS-treated mice (Fig. 1B). In addition, IFN-γ treatment promoted significant suppression of disease in chronic progressive EAE mice (NOD mice) after 10 days of treatment (Fig. 1C).

**Fig. 2:**
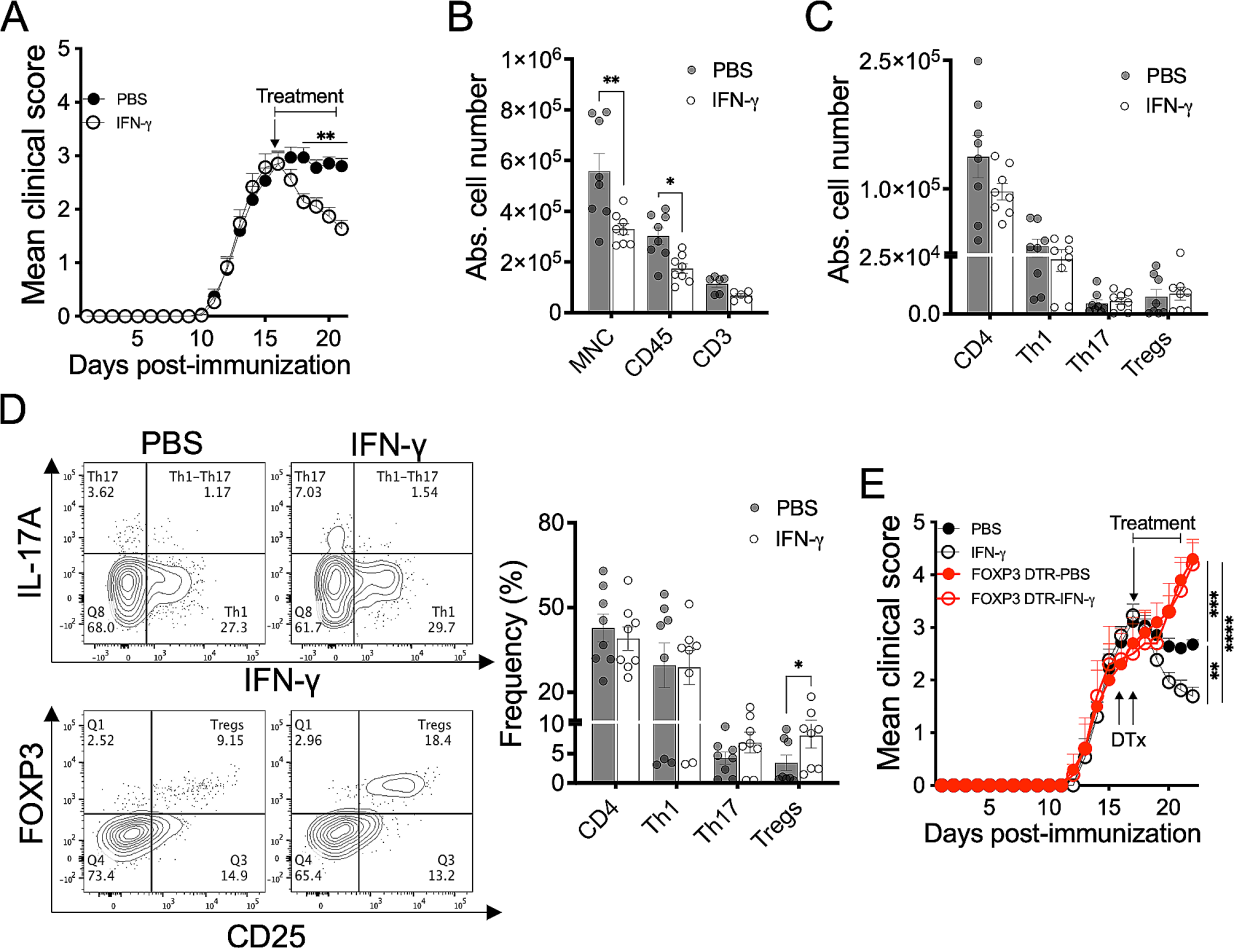
Therapeutic effect of IFN-γ depends on the presence of FOXP3^+^ Treg cells in EAE. (**A**) Mice induced with chronic EAE were treated daily with 1 µg IFN-γ (empty black circles) or PBS (solid black circles) at the peak of disease for five days (*n* = 10/group, mean of three independent experiment). (**B**-**D**) At the end of treatment, spinal cords were isolated and the absolute number of (**B**) mononuclear cells (MNC), CD45^+^ cells, and CD3^+^ cells, and (**C**) the absolute number and (**D**) frequency of CD4^+^ T cells, Th1, Th17 and Treg cells were determined by flow cytometry. (**E**) Wild type (*n* = 13/group) and FOXP3-DTR mice (*n* = 5/group) were induced with EAE and treated with 1 µg IFN-γ (empty black and red circles) or PBS (solid black and red circles) at the peak of disease for five days. To deplete FOXP3^+^ Treg cells, FOXP3-DTR mice were i.p. treated with one dose 2.5 µg of diphtheria toxin (DTx) two days before starting IFN-γ treatment and with another similar dose two days later. Three independent experiments were pooled. Results are shown as the mean ± SEM. Data were analyzed using two-way ANOVA followed by Bonferroni post-hoc test (**A**), Mann–Whitney *U* test (**B**-**D**), or one-way ANOVA followed by Bonferroni post-hoc test (**E**). *p < 0.05, **p < 0.01, ***p < 0.001, ****p < 0.0001

Next, we investigated the impact of IFN-γ treatment on peripheral and CNS-infiltrating CD4^+^ T cell subpopulations in chronic MOG_35 − 55_ peptide EAE in B6 mice. Spinal cords isolated from EAE mice treated with IFN-γ for 5 days (Fig. 2A) exhibited a significant reduction in the number of mononuclear cells (MNC) and infiltrating CD45^+^ cells compared with those from PBS-treated EAE mice (Fig. 2B). There was no statistical difference in the absolute number of MNC and CD45^+^ cells in brains from IFN-γ and PBS-treated EAE mice and similar numbers of cells were found in spleen and LN from both groups of EAE mice (Supplementary Fig. 2A). Lower absolute numbers of CD3^+^ T cells were observed in IFN-γ-treated EAE mice compared to PBS-treated EAE mice, but this difference did not reach statistical significance (Fig. 2B). Analysis of the impact of IFN-γ on different subpopulations of CD4^+^ T lymphocytes (Supplementary Fig. 1A and B) showed a lower absolute number of CD4^+^ T cells and Th1 cells in spinal cords from IFN-γ-treated EAE mice than in control EAE mice, but these differences were not significant. The number of Th17 and Treg cells was similar between both groups of mice (Fig. 2C). The absence of impact on Th17 and Treg cell number led us to determine if the frequencies might be modulated by IFN-γ treatment. Similar frequencies of CD4^+^ T cells and Th1 cells and a slight but not significant increase of Th17 cells were determined in spinal cords from IFN-γ and PBS-treated EAE mice. Notably, a significantly higher frequency of Treg cells was found in spinal cords from IFN-γ-treated EAE mice (Fig. 2D). No significant differences in numbers or frequencies of CD4^+^ T cell subpopulations were observed in the brain and secondary lymphoid organs (cervical/axillary draining LN and spleen); although the absolute number of Treg cells tended to be higher in brains and spleens of IFN-γ-treated EAE mice (Supplementary Fig. 2B). These results led us to examine the functional requirement of Treg cells in mediating the protective effect of IFN-γ in EAE. To do that, we used the transgenic FOXP3-DTR mouse model, which allows the conditional depletion of FOXP3^+^ Treg cells after systemic administration of diphtheria toxin (Supplementary Fig. 2C). Consistent with a previous study [21], depletion of FOXP3^+^ Treg cells at the peak of EAE induced a more severe disease over time. Interestingly, EAE mice depleted of Treg cells were refractory to the protective effects of IFN-γ, suggesting that functional Treg cells are required at the time of IFN-γ treatment for effective therapeutic regulation of EAE (Fig. 2E).

### IFN-γ does not exert a direct effect on Treg cell differentiation

**Fig. 3:**
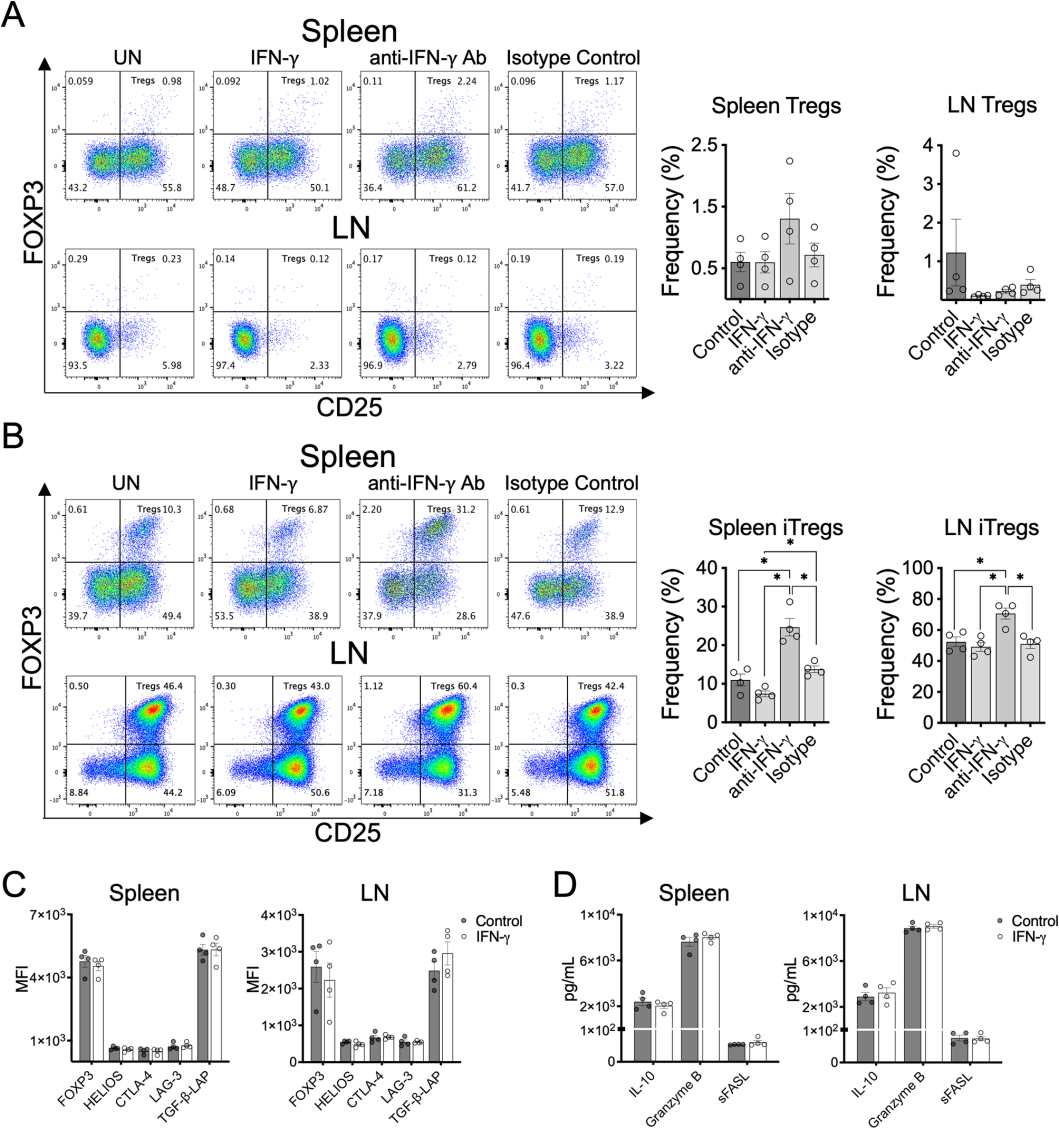
IFN-γ does not exert a direct effect on Treg cell differentiation. (**A**) CD4^+^CD25^−^ T cells isolated (purity > 90%) from spleen or draining lymph nodes (LN) of EAE mice at the peak of disease were stimulated for 3 days with 2 µg/mL plate-bound anti-CD3 and 1 µg/mL soluble anti-CD28 in the absence (control) or presence of either 25 ng/mL IFN-γ, 5 µg/mL anti-IFN-γ antibody or 5 µg/mL isotype control antibody. (**B**) Similar assay as described in (**A**) in the presence of 2 ng/mL TGF-β and 10 ng/mL IL-2 to promote differentiation of inducible Treg cells (iTregs). The frequency of Treg cells (CD3^+^CD4^+^CD25^+^FOXP3^+^) was determined by flow cytometry. (**C**) Purified fraction of CD4^+^CD25^+^ cells (containing > 82% of Treg cells) obtained during the purification of CD4^+^CD25^−^ T cells were stimulated with 2 µg/mL plate-bound anti-CD3 and 1 µg/mL soluble anti-CD28 for 3 days in the absence (control, solid gray bars) or presence of 25 ng/mL IFN-γ (empty gray bars). The expression of regulatory markers FOXP3, HELIOS, CTLA-4, LAG-3 and TGF-β-LAP was determined as median fluorescence intensity (MFI) by flow cytometry. (**D**) Secretion of IL-10, Granzyme B and sFASL in cell culture supernatants was assessed by multiplex assay. Results are shown as the mean ± SEM (*n* = 4). Data were analyzed using Mann–Whitney *U* test. *p < 0.05

In order to determine whether IFN-γ might directly trigger the conversion of CD4^+^CD25^−^ T cells into Treg cells, CD4^+^CD25^−^ T cells were purified from spleen and draining LN of EAE mice at the peak of the disease (> 90% purity, Supplementary Fig. 3A) and in vitro activated with anti-CD3 and anti-CD28 antibodies with IFN-γ or anti-IFN-γ antibody for 72 h. Different concentrations of IFN-γ (range between 1 and 100 ng/ml) did not induce conversion of CD4^+^CD25^−^ T cells isolated from spleen or LN of EAE mice into Treg cells (Fig. 3A and Supplementary Fig. 3B). Next, we analyzed whether IFN-γ might have a direct effect on the generation of inducible Treg cells (iTreg) promoted by TGF-β and IL-2. The results showed that addition of IFN-γ did not change the frequency of iTreg cells starting from CD4^+^CD25^−^ T cells isolated from spleens or LN (Fig. 3B). Moreover, addition of blocking antibodies to IFN-γ led to significantly higher generation of iTreg cells compared to untreated and IFN-γ-stimulated CD4^+^CD25^−^ T cells (Fig. 3B). We also determined whether IFN-γ might have a direct effect on the fraction of CD4^+^CD25^+^ T cells obtained during the purification of CD4^+^CD25^−^ T cells (> 89% of total FOXP3^+^ cells - Supplementary Fig. 3A). IFN-γ treatment did not affect the expression of regulatory markers associated with Treg cells such as FOXP3, CTLA-4, HELIOS, LAG-3, and TGF-β-LAP (Fig. 3C) or the secretion of IL-10, Granzyme B, and soluble FAS ligand (Fig. 3D) in CD4^+^CD25^+^ T cells in vitro activated with anti-CD3 and anti-CD28 antibodies. Taken together, these results indicate that IFN-γ does not directly promote the generation of Treg cells, but does, in fact, inhibits the differentiation of iTreg cells.

### Therapeutic effect of IFN-γ is dependent on TGF-β and PD-1

**Fig. 4:**
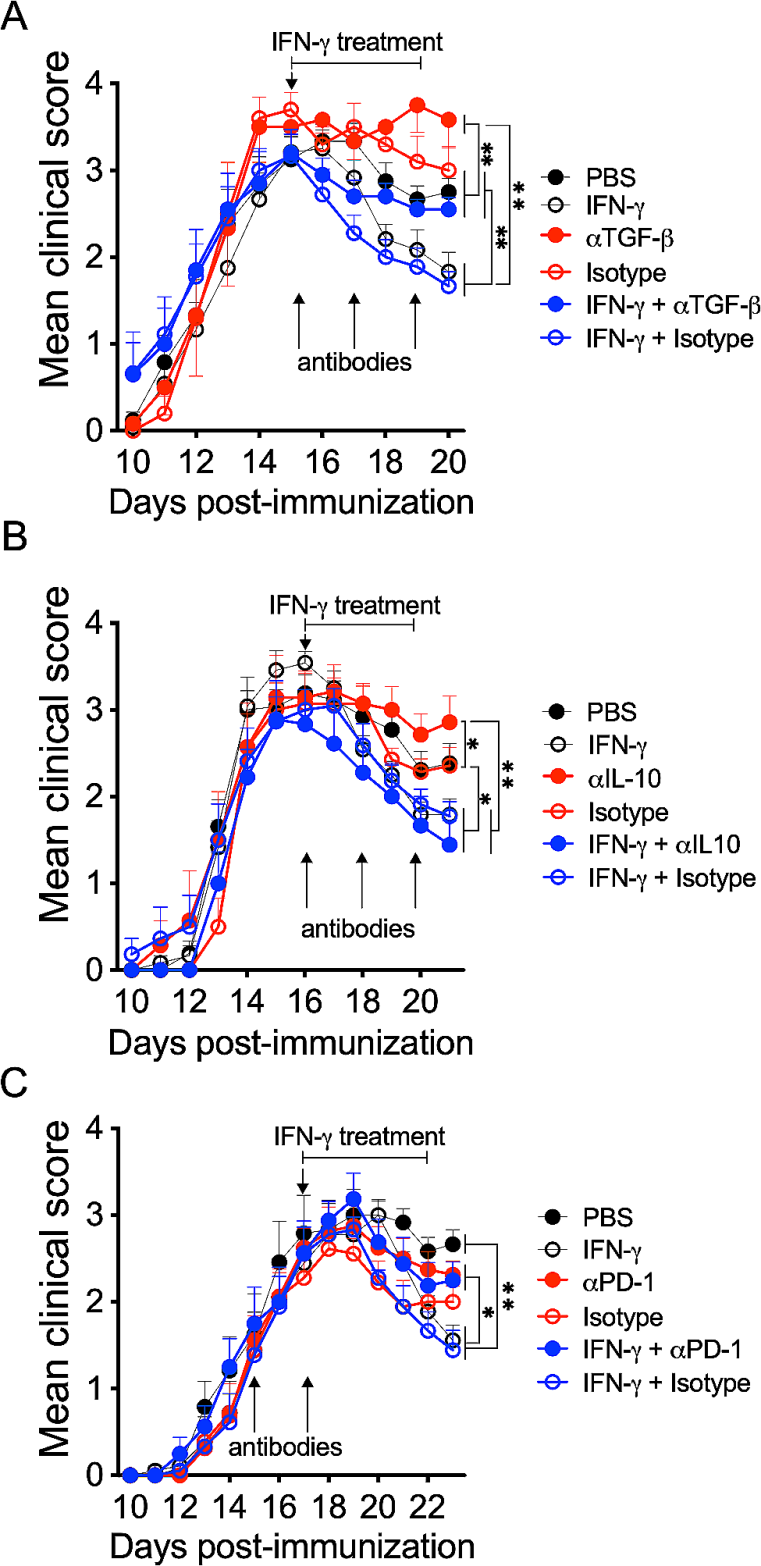
Therapeutic effect of IFN-γ is dependent on TGF-β and PD-1. EAE mice were treated at the peak of disease for 5 days with 1 µg/day IFN-γ alone (empty black circles) or along with (**A**) 100 µg of neutralizing antibody against TGF-β (solid blue circles, *n* = 6) or (**B**) IL-10 (solid blue circles, *n* = 7) administered at days 0, 2 and 4 after IFN-γ treatment, or with (**C**) 500 µg neutralizing anti-PD-1 antibody (solid blue circles, *n* = 8) administered two days before starting IFN-γ treatment and at the same time of IFN-γ treatment. Control EAE mice were treated with PBS (vehicle, solid black circles) or corresponding isotype antibody alone (empty red circles) or along with IFN-y (empty blue circles). Two independent experiments were pooled. Results are shown as the mean ± SEM. Data were analyzed using one-way ANOVA followed by the Bonferroni post-hoc test. *p < 0.05, **p < 0.01

Because IFN*-*γ has no direct effect on the induction of Treg cells, we asked if this cytokine might modulate regulatory molecules involved in the differentiation and activity of FOXP3^+^ Treg cells, such as TGF-β [46], IL-10 [47], and PD-1 [16]. As expected, in vivo blockade of either TGF-β or IL-10 in untreated (without IFN-γ) EAE mice slightly exacerbated disease severity compared to control groups. Interestingly, neutralization of TGF-β, but not IL-10, interfered with the protective effects of IFN-γ on EAE (Fig. 4A and B). Similarly, disease amelioration induced by IFN-γ was prevented when EAE mice were administered blocking antibodies to PD-1 (Fig. 4C). Based on these results, we hypothesized that IFN-γ might have an indirect role on FOXP3^+^ Treg cells regulating the expression of TGF-β and ligands of PD-1 in APC.

### IFN-γ/STAT1 axis induces a subset of splenic CD11b^+^ myeloid cells expressing PD-L1 and TGF-β in EAE mice

**Fig. 5:**
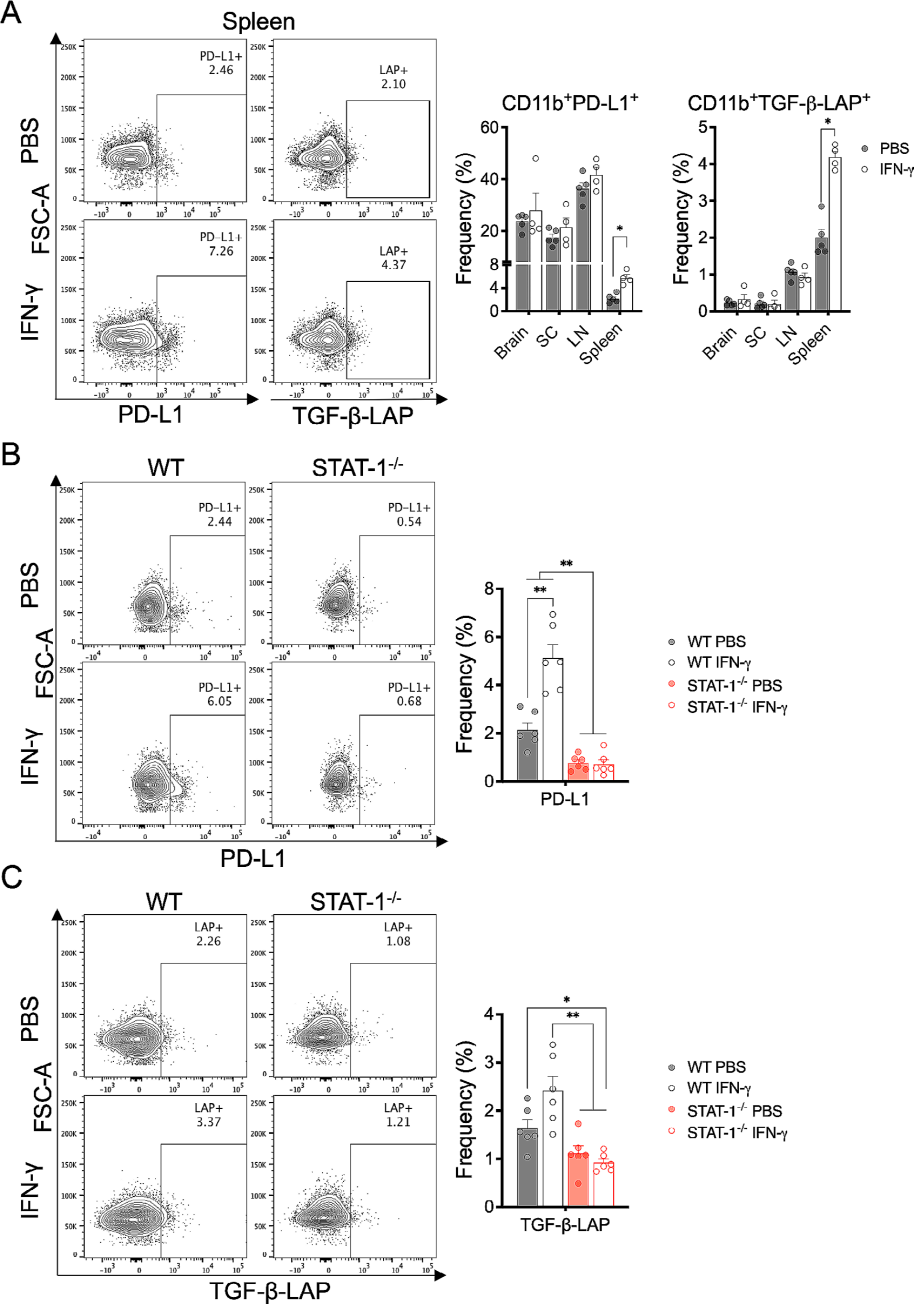
IFN-γ/STAT-1 axis induces splenic CD11b^+^ myeloid cells expressing PD-L1 and TGF-β-LAP. (**A**) Frequency of PD-L1^+^ and TGF-β-LAP^+^ CD11b^+^ cells in brain, spinal cord (SC), draining lymph nodes (LN) and spleen from EAE mice treated at the peak of disease with IFN-γ (empty gray bars, *n* = 4) or PBS (solid gray bars, *n* = 5) for 5 days was determined by flow cytometry. (**B**) Frequency of CD11b^+^PD-L1^+^ cells and (**C**) CD11b^+^TGF-β-LAP^+^ cells were determined by flow cytometry in spleen from wild-type mice (solid and empty gray bars, *n* = 6) and STAT-1 deficient mice (solid and empty red bars, *n* = 6) induced with EAE and treated at the peak of disease with IFN-γ (empty bars) or PBS (vehicle, solid bars) for 5 days. Three independent experiments were pooled. Results are shown as the mean ± SEM. Data were analyzed using Mann–Whitney *U* or Kruskal-Wallis test. *p < 0.05, **p < 0.01

To address the above hypothesis, we determined the impact of IFN-γ on myeloid APC in EAE by analyzing the frequency of CD11b^+^ myeloid cells expressing PD-L1 and TGF-β-LAP in brain, spinal cord, spleen, and LN from EAE mice. The results showed that the frequency of CD11b^+^ cells expressing PD-L1 and TGF-β-LAP was significantly higher in the spleen from IFN-γ-treated EAE mice than in those from PBS-treated EAE mice. There was no difference in other organs (Fig. 5A).

The biological activity of IFN-γ is mainly mediated through STAT-1, thus we determined whether the in vivo modulation of splenic CD11b^+^ myeloid cells by IFN-γ is also dependent on STAT-1. We found that the enhanced frequency of CD11b^+^ cells expressing PD-L1 and TGF-β-LAP induced by IFN-γ was significantly reversed in EAE mice lacking STAT-1 (Fig. 5B and C). Therefore, these results indicate that the IFN-γ-STAT-1 axis mediates protective effects in EAE via inducing a subset of splenic CD11b^+^ myeloid cells expressing PD-L1 and TGF-β-LAP.

### IFN-γ acts directly on splenic CD11b^+^ cells inducing a tolerogenic phenotype and function

**Fig. 6:**
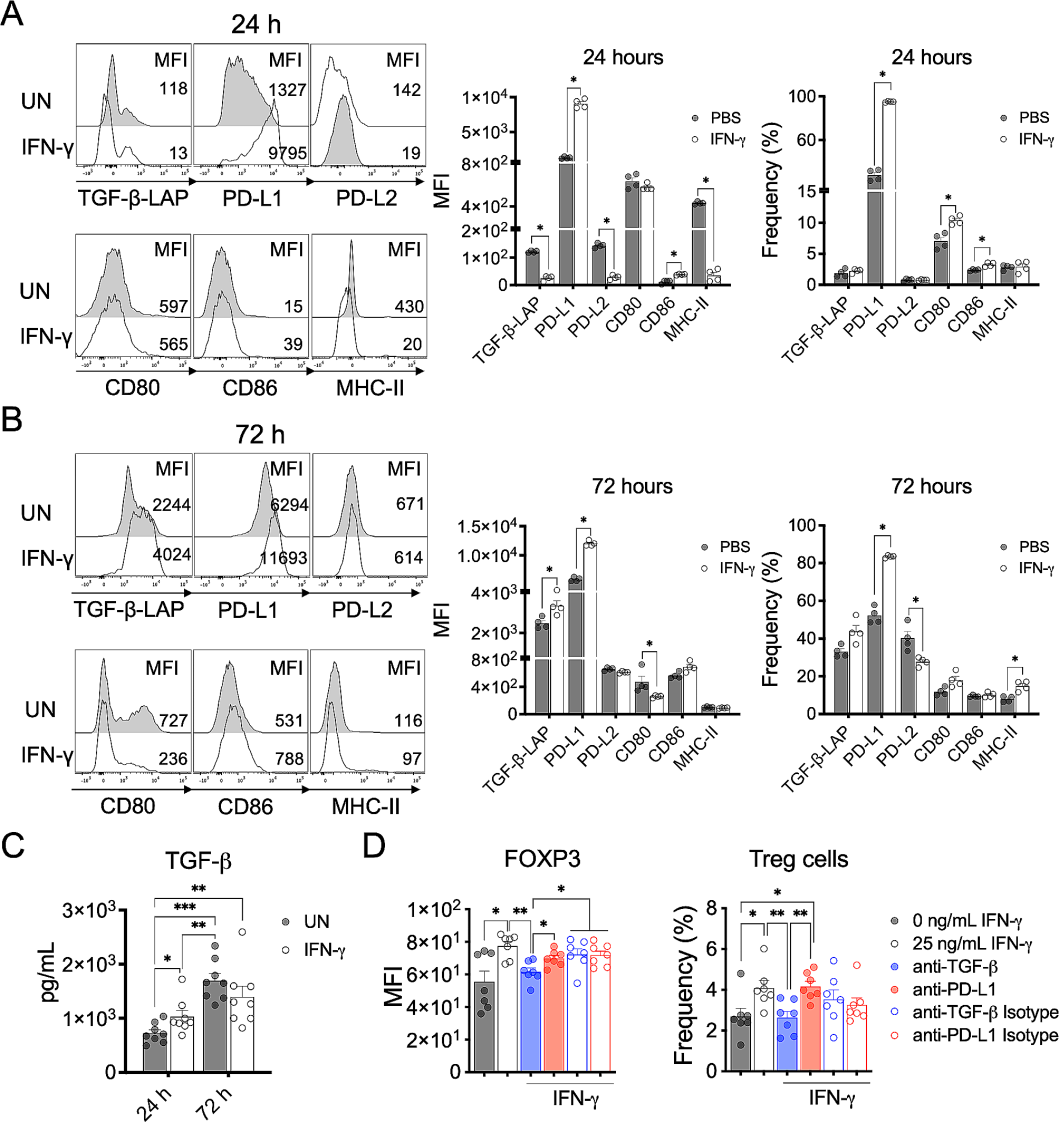
IFN-γ directly induces a tolerogenic phenotype and function in splenic CD11b^+^ cells from EAE mice. Splenic CD11b^+^ cells isolated from EAE mice were in vitro stimulated with 25 ng/mL IFN-γ (empty gray bars) or left untreated (UN, solid gray bars) for (**A**) 24 and (**B**) 72 h in the presence of 10 µg/mL MOG_33 − 55_ peptide. Frequency and median fluorescence intensity (MFI) of TGF-β-LAP, PD-L1, PD-L2, CD80, CD86 and MHC-II were analyzed by flow cytometry (*n* = 4). (**C**) Cell culture supernatants were collected at 24 and 72 h and concentration of TGF-β1 was measured by multiplex assay (*n* = 8). (**D**) Splenic CD11b^+^ cells from EAE mice were preconditioned with 10 µg/mL MOG_33 − 55_ peptide and 25 ng/mL IFN-γ for 24 h, washed, and co-cultured with naïve CD4^+^ T cells (ratio 1:1) from healthy mice in the presence of 1 µg/mL soluble anti-CD3 antibody and with either anti-TGF-β antibody (solid blue bar), anti-PD-L1 antibody (solid red bar), or corresponding isotype control antibodies (empty blue and red bars). After 72 h, the frequency of Treg cells (CD4^+^CD25^+^FOXP3^+^) was determined by flow cytometry. Results are shown as the mean ± SEM. Data were analyzed using Mann–Whitney *U* test. *p < 0.05, **p < 0.01

Next, we wanted to determine whether IFN-γ might directly regulate the expression of PD-L1, TGF-β-LAP, and other molecules involved in T cell activation on CD11b^+^ myeloid cells. CD11b^+^ cells isolated from spleen (> 87% purity, Supplementary Fig. 4) at peak EAE were stimulated with IFN-γ and MOG_35 − 55_ peptide for 24–72 h and the expression of TGF-β-LAP, co-inhibitory molecules (PD-L1 and PD-L2), co-stimulatory molecules (CD80 and CD86), and MHC-II molecules was evaluated by flow cytometry. The levels of secreted TGF-β were assessed in the cell culture supernatant by multiplex assay. The results showed that CD11b^+^ cells treated with IFN-γ for 24 h presented a significantly lower expression of TGF-β-LAP on the cell surface, as measured by mean fluorescence intensity (MFI), which was associated with a higher secretion of TGF-β1 compared to unstimulated cells (Fig. 6A and C); suggesting an enhanced processing and secretion of TGF-β in response to IFN-γ in these cells. After 72 h, IFN-γ-treated CD11b^+^ cells showed a significantly higher expression of TGF-β-LAP^+^ on the cell surface but a similar secretion of TGF-β than untreated CD11b^+^ cells (Fig. 6B and C). Given that it has been reported that IL-27, a cytokine induced by IFN-γ, is involved in antigen-specific immune tolerance in EAE by inducing PD-L1 expression in dendritic cells and production of IL-10 in CD4^+^ T cells [48, 49], we also determined the levels of IL-27 and IL-10 in the cell culture supernatant. The results showed that splenic CD11b^+^ cells from EAE mice secreted low levels of IL-27 and IL-10 and that they were not induced by IFN-γ after 24 and 72 h of treatment (Supplementary Fig. 5A). Remarkably, stimulation with IFN-γ for 24 h and 72 h induced a significantly higher frequency of CD11b^+^ cells expressing PD-L1 as well as a higher expression of PD-L1 compared to untreated CD11b^+^ cells (Fig. 6A and B). The expression of PD-L2 on CD11b^+^ cells was significantly lower in IFN-γ-treated CD11b^+^ cells than untreated cells at 24 h, and the frequency of CD11b^+^ cells expressing PD-L2 was also significantly reduced after 72 h.

The frequency of CD11b^+^ cells expressing CD80 and CD86 was initially increased at 24 h by IFN-γ but decreased to control levels after 72 h. The expression of CD80 was significantly lower in response to IFN-γ after 72 h (Fig. 6A and B). Although there was no difference in the frequency of CD11b^+^ cells expressing MHC-II molecules, CD11b^+^ cells stimulated with IFN-γ showed significantly lower expression of MHC-II molecules at 24 h compared to untreated CD11b^+^ cells (Fig. 6A). After 72 h, IFN-γ induced a significantly higher frequency of CD11b^+^MHC-II^+^ cells but with a similar level of expression of MHC-II molecules than control cells (Fig. 6B). Taken together, these results suggest that IFN-γ acts directly on CD11b^+^ cells regulating the expression of molecules involved in immune tolerance resulting in an early secretion of TGF-β1, enhanced expression of PD-L1 and CD86, and decreased expression of MHC-II molecules, followed by an increased expression of TGF-β-LAP and PD-L1, and a reduced expression of CD80.

Therefore, we next investigated the capability of IFN-γ-treated CD11b^+^ cells to induce differentiation of Treg cells. CD11b^+^ cells isolated from spleen at peak EAE were cultured in the presence of IFN-γ and MOG_35 − 55_ peptide for 24 h, washed, and then co-cultured with naïve CD4^+^ T cells from healthy mice (1:1 ratio) and soluble anti-CD3 antibody for 72 h. The expression of FOXP3^+^ (MFI) in CD4^+^ T cells and the frequency of Treg cells (CD4^+^CD25^+^FOXP3^+^) was significantly higher in naïve CD4^+^ T cells co-cultured with IFN-γ-stimulated CD11b^+^ cells than with untreated CD11b^+^ cells (Fig. 6D and Supplementary Fig. 5B). Interestingly, neutralization of TGF-β, but not of PD-L1, significantly decreased the expression of FOXP3^+^ in CD4^+^ T cells and the frequency of Treg cells to a similar level of untreated cells. These results indicate that IFN-γ induces tolerogenic splenic CD11b^+^ cells with the capability to convert naïve CD4^+^ T cells into Treg cells.

### Transfer of CD11b^+^ myeloid cells from IFN-γ-treated EAE mice to untreated EAE mice induces disease amelioration by reducing CNS-infiltrating helper T cells

**Fig. 7:**
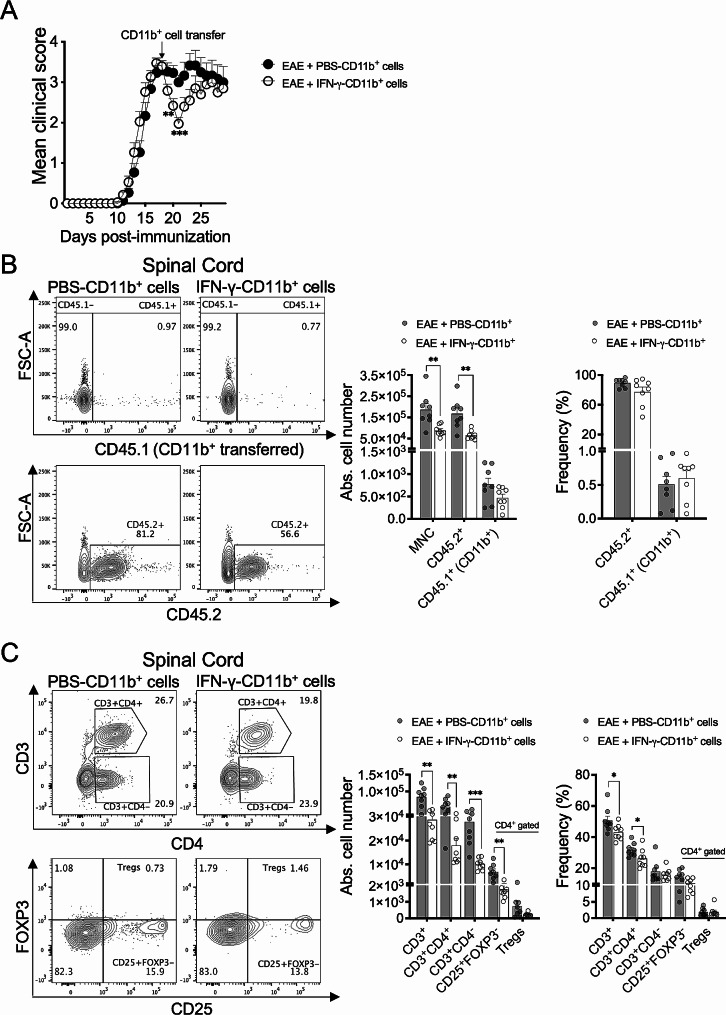
Adoptive transfer of splenic CD11b^+^ cells from IFN-γ-treated EAE mice into recipient EAE mice suppresses disease progression. (**A**) Splenic CD11b^+^ cells (10^6^) from CD45.1 EAE mice treated for 5 days with IFN-γ (empty black circles) or PBS (solid black circles) were i.v. transferred into CD45.2 EAE mice at the peak of disease (*n* = 15). Clinical symptoms were monitored daily. Four independent experiments were pooled. (**B**-**C**) After three days of transfer, mononuclear cells from spinal cords of recipient EAE mice receiving CD45.1^+^CD11b^+^ cells from EAE mice treated with IFN-γ (empty gray bars, *n* = 8) or PBS (solid gray bars, *n* = 8) were isolated and absolute number and frequencies of (**B**) mononuclear cells (MNC), CD45.2^+^ cells, and CD45.1^+^CD11b^+^ cells, and (**C**) CD3^+^ T cells, CD3^+^CD4^+^ T cells, CD3^+^CD4^−^ T cells, CD3^+^CD4^+^CD25^+^FOXP3^−^ T cells, and Treg cells (CD3^+^CD4^+^CD25^+^FOXP3^+^), were determined by flow cytometry (*n* = 8, two different experiments were pooled). Results are shown as the mean ± SEM. Data were analyzed using two-way ANOVA followed by the Bonferroni post-hoc test (**A**) or Mann–Whitney *U* test (**B**-**C**). *p < 0.05, **p < 0.01, ***p < 0.001

To demonstrate the therapeutic and tolerogenic potential of splenic CD11b^+^ myeloid cells induced by IFN-γ, these cells were isolated from IFN-γ or PBS-treated EAE mice (CD45.1) and transferred to untreated EAE mice (CD45.2) at the peak of EAE. EAE mice receiving a single injection of splenic CD11b^+^ myeloid cells from IFN-γ-treated EAE mice exhibited a significant and rapid amelioration of clinical symptoms followed by a relapse of disease compared to control EAE mice receiving CD11b^+^ cells from PBS-treated EAE mice (Fig. 7A). After three days of transfer, the total number of mononuclear cells and CD45.2^+^ cells were significantly lower in spinal cord from EAE mice receiving splenic CD11b^+^ cells from IFN-γ-treated EAE mice than those receiving splenic CD11b^+^ myeloid cells from PBS-treated EAE mice (Fig. 7B). Instead, the absolute cell number and frequency of CD45.1^+^CD11b^+^ cells in spinal cord were not significantly different between both groups of recipients EAE mice (Fig. 7B). There was also no difference in the absolute cell number and frequency of CD45.2^+^ cells and CD45.1^+^CD11b^+^ cells in the spleen from both groups of recipients EAE mice (Supplementary Fig. 6A). Interestingly, spinal cords from EAE mice receiving CD11b^+^ myeloid cells from IFN-γ-treated EAE mice exhibited a significantly lower absolute number of CD3^+^ T cells, CD3^+^CD4^+^ T helper cells, CD3^+^CD4^−^ cells, and CD25^+^FOXP3^−^ cells compared to spinal cords from EAE mice receiving CD11b^+^ myeloid cells from PBS-treated EAE mice. Likewise, the absolute number of Treg cells was lower in EAE mice receiving CD11b^+^ myeloid cells from IFN-γ-treated EAE mice; but it did not reach statistical significance (Fig. 7C). Although reduction in all these cell populations might be influenced by the lower absolute numbers of total mononuclear cells observed in spinal cord from EAE mice receiving CD11b^+^ myeloid cells from IFN-γ-treated EAE mice (Fig. 7B), the frequency of CD3^+^ T cells and CD3^+^CD4^+^ helper T cells was also significantly lower in recipients of CD11b^+^ myeloid cells from IFN-γ-treated EAE mice (Fig. 7C). There was no difference in the absolute cell number and frequency of all T cell subpopulations analyzed in spleen from both groups of recipients EAE mice (Supplementary Fig. 6B). Taken together, these results indicate that adoptive transfer of splenic CD11b^+^ myeloid cells from IFN-γ-treated EAE mice to recipient (untreated) EAE mice suppresses disease progression by limiting CNS-infiltrating effector helper T cells.

## Discussion

IFN-γ is primarily considered a pro-inflammatory cytokine based on its key role against infections and tumors as well as the promotion of Th1 cell-driven inflammation. However, cumulative evidence has revealed that IFN-γ also exerts protective effects in EAE (reviewed in [35, 36, 42, 44]). Here, we provide further support for these findings, demonstrating that IFN-γ limits the magnitude of new relapses in RR-EAE and slows clinical course in chronic progressive EAE. Also, beneficial effects of IFN-γ have been reported in other murine models of transplantation, asthma, and autoimmune diseases such as collagen-induced arthritis (CIA), experimental autoimmune uveitis (EAU), autoimmune nephritis, and autoimmune myocarditis [50–58]. More recently, we have shown that IFN-γ is essential for maintaining immune tolerance to aquaporin-4 (AQP4) antigen and that its absence leads to autoimmune inflammation and severe clinical disease resembling AQP4-IgG^+^ neuromyelitis optica spectrum disorders (NMOSD) [59].

Treg cells play a critical role in maintaining immune tolerance in EAE and MS [60]. Indeed, FOXP3^+^-depleted EAE mice exhibit worsening of disease associated with a significant increase in CNS-infiltrating CD4^+^ T effector cells [21]. In addition, several reports have shown that there is a dysregulation in the Treg cell population in both MS and EAE due to either a reduction in their frequency [61, 62], or in their functionality [18, 25, 63]. In this study, we found that although the therapeutic activity of IFN-γ in EAE is dependent on the presence of functional Treg cells, IFN-γ does not trigger direct differentiation of FOPX3^+^ Treg cells and; on the contrary, in vitro neutralization of IFN-γ induces an increase in their cell number. Consistent with our findings, several studies have shown that IFN-γ inhibits the in vitro and in vivo development of Treg cells and that the neutralization of IFN-γ markedly promotes the differentiation and suppressive function of iTregs [64–67]. Furthermore, some reports have included antibodies against IFN-γ to improve in vitro differentiation of iTreg cells [65, 68]. In contrast, Wang et al., reported that IFN-γ can directly induce in vitro FOXP3 expression in murine TCR-stimulated CD4^+^CD25^−^ T cells and that their transfer to EAE recipient mice reduced clinical symptoms. Furthermore, IFN-γ induced, in a dose-dependent manner, increased expression of FOXP3 in CD4^+^CD25^−^ T cells from healthy individuals and the resulting Treg cells inhibited autologous T cell proliferation [45]. Similarly, IFN-γ promoted a significant increase in the conversion of TCR-stimulated CD4^+^CD25^−^ T cells to suppressive CD4^+^CD25^+^FOXP3^+^ T cells in patients with Guillain-Barré syndrome or myasthenia gravis [69, 70]. Another study showed that differentiation of iTreg cells starting from healthy human CD4^+^CD45RA^+^ T cells in the presence of IFN-γ exhibited higher expression of FOXP3 than iTreg cells differentiated only with TGF-β and both TGF-β-induced iTreg cells and IFN-γ-boosted iTreg cells had similar suppressive activity [71]. Therefore, the role of IFN-γ in the conversion of CD4^+^CD25^−^ T cells to Treg cells is controversial. The difference between these results might be explained by the microenvironment from which CD4^+^ T cells were isolated. Wang et al. [45], generated FOXP3^+^ Treg cells from CD4^+^CD25^−^ T cells obtained from the spleen of unprimed (without EAE) mice. In this study, we used CD4^+^CD25^−^ T cells obtained from LN and spleens of EAE mice. In addition, the conversion rate of CD4^+^CD25^−^ T cells into CD4^+^CD25^+^FOXP3^+^ T cells was significantly higher in healthy individuals than in patients with Guillain-Barré syndrome or myasthenia gravis [69, 70]. Therefore, the response of CD4^+^CD25^−^ T cells to IFN-γ may differ between patients and healthy subjects and between patients in different stages of disease. In the same way, the inflammatory microenvironment might also affect the responsiveness of Treg cells to signals promoting suppressive activity, such as IFN-γ. Supporting this notion, it has been reported that FOXP3^+^ Treg cells readily accumulate in the CNS of EAE mice, but they are unable to inhibit MOG_35 − 55_ peptide-specific effector T cells derived from the CNS at the peak of inflammation due to their production of IL-6 and TNF [18, 72].

It has been reported that RRMS patients exhibit a significantly higher percentage of Treg cells producing IFN-γ than healthy individuals. Furthermore, the in vitro suppressive activity of IFN-γ^+^FOXP3^+^ Treg cells from RRMS patients was significantly lower than that from healthy controls. Besides, blocking IFN-γ significantly increased the suppressive activity of IFN-γ^+^FOXP3^+^ Treg cells from RRMS patients, but not to the same level of suppressive activity as IFN-γ^+^FOXP3^+^ Treg cells from healthy subjects [66]. Therefore, these results suggest that the dual role of IFN-γ on the differentiation and tolerogenic function of Treg cells might be determined by the conditions of the particular inflammatory microenvironment. Indeed, we and other investigators have found that the opposite roles of IFN-γ in EAE are dependent on the stage of disease: pathogenic during the induction phase of EAE, but beneficial during the early and chronic effector phases [39, 42–44, 73].

Our results show that the therapeutic activity of IFN-γ in EAE also depends on TGF-β and PD-1. PD-1 is a co-inhibitory surface receptor that plays a pivotal role in regulating T cell activation. The interaction between PD-1 and its ligands PD-L1 and PD-L2 regulates peripheral CD4^+^ and CD8^+^ T cell tolerance attenuating self-reactive T cell response and promoting Treg cell differentiation and function [16]. In particular, expression of PD-L1 is regulated by IFN-γ and this ligand has a critical regulatory role in EAE [16, 74–78]. In turn, TGF-β has been described as one of the most essential cytokines involved in Treg cell development and function as well as a key factor regulating EAE development [46, 79–81]. Interestingly, Treg cell conversion induced by splenic DCs in a tumor microenvironment is dependent on TGF-β and PD-L1 signaling [82]. In addition, Casella et al. [49], have described that antigen-specific tolerance induction by intravenous injection of high doses of autoantigen halts EAE progression by inducing PD-L1 expression in CNS monocyte-derived DCs via IFN-γ/IL-27-dependent mechanism. Based on this evidence, we hypothesized that IFN-γ might have an indirect role on FOXP3^+^ Treg cells by inducing expression of TGF-β and ligands of PD-1 in APC. In line with our hypothesis, we found that IFN-γ induces an increased frequency of a subset of splenic CD11b^+^ cells expressing PD-L1 and TGF-β-LAP^+^ in EAE. Furthermore, our ex vivo assays show that IFN-γ acts directly on CD11b^+^ cells inducing a tolerogenic phenotype characterized by an early secretion of TGF-β1, enhanced expression of PD-L1, and decreased expression of MHC-II molecules, followed by an increased expression of TGF-β-LAP and PD-L1, and a reduced expression of CD80. IL-27 and IL-10 are not induced by IFN-γ and they would not be involved in the tolerogenic mechanism mediated by IFN-γ. Consistently, in vivo neutralization of IL-10 does not interfere with the protective effects of IFN-γ on EAE. Notably, IFN-γ-treated CD11b^+^ cells promote antigen-specific conversion of CD4^+^ T cells into Treg cells through the secretion of TGF-β. Because PD-L1 was not involved in this process, it is not ruled out that the role of PD-1 in EAE is partially independent of Treg induction. In this case, it is plausible that the role of PD-1 is rather related with attenuation of self-reactive T cell response [16]. Supporting our results showing that the beneficial activity of IFN-γ is dependent on TGF-β, we have recently found that primary myeloid cells/microglia cultures obtained from spinal cord of IFN-γ-treated EAE mice that were ex vivo re-stimulated with low dose (1 ng/ml) IFN-γ and MOG_35 − 55_ peptide showed significantly enhanced tolerogenic activity, promoting induction of CD4^+^ Treg cells associated with increased TGF-β secretion [44]. Additionally, it has been reported that IFN-γ signaling is required for tolerogenic function of DCs mediated by TGF-β in EAE [83], and that IFN-γ along with TNF-α produced by T cells in combination with TGF-β and B7 molecules expressed by neurons are critical in the generation of neuron-induced Treg cells in the CNS of EAE mice [84]. Collectively, this evidence supports the ability of IFN-γ to endow different cell types with tolerogenic properties.

Remarkably, splenic CD11b^+^ myeloid cells from IFN-γ-treated EAE mice showed a significant therapeutic potential in that intravenous injection of these cells in untreated EAE mice induced a rapid and significant amelioration of clinical symptoms. Disease attenuation induced by adoptive transfer of splenic CD11b^+^ myeloid cells from IFN-γ-treated EAE mice was associated with lower CNS infiltration of mononuclear cells and effector CD3^+^CD4^+^ helper T cells. Although there was no significant difference in Treg cells at the time of evaluation (3 days after injection), it does not rule out the possibility that Treg cells are induced by splenic CD11b^+^ myeloid cells earlier and/or transitorily. Further experiments will be necessary to identify which type of APC mediates the therapeutic effects of splenic CD11b^+^ myeloid cells. A possible cellular target of IFN-γ might be DCs because transfer of healthy splenic DC ex vivo stimulated with IFN-γ significantly suppressed clinical symptoms of EAE and prevented diabetes onset in NOD mice [49, 85, 86].

We found that that the induction of splenic CD11b^+^ myeloid cells expressing PD-L1 and LAP-TGF-β depends on STAT-1, an essential transcriptional factor involved in the maintenance of immunological self-tolerance and function of Treg cells in EAE [87, 88]. Interestingly, we have recently reported that amelioration of EAE symptoms and induction of homeostatic microglia by IFN-γ is also dependent on STAT-1. Therefore, our previous and current results reveal a novel role of the IFN-γ/STAT-1 axis in the induction of tolerogenic microglia and splenic CD11b^+^ myeloid cells involved in the suppression of EAE development and shed light on the protective mechanism of IFN-γ showing that it induces tolerogenic activity in EAE targeting both peripheral and CNS-resident APC.

## Conclusions

Collectively, our results show that the protective mechanisms of IFN-γ in EAE depend on the presence of FOXP3^+^ Treg cells. However, IFN-γ does not exert a direct effect on Treg cells but rather induces a subset of splenic CD11b^+^ myeloid cells with tolerogenic phenotype and function able to induce conversion of naïve CD4^+^ T cells into Treg cells signaling through STAT-1 and promoting TGF-β secretion. Furthermore, IFN-γ-induced splenic CD11b^+^ myeloid cells exert therapeutic activity in recipient EAE mice limiting CNS infiltration of mononuclear cells and helper T cells. Therefore, our findings reveal a previously unknown protective mechanism of IFN-γ in a neuroinflammatory context and contribute to clarifying its paradoxical role in EAE and MS.

### Electronic supplementary material

Below is the link to the electronic supplementary material.

Supplementary Material 1

## Data Availability

The data supporting the findings of this work are available upon reasonable request.

## Abbreviations

APC: antigen presenting cells
CD: cluster of differentiation
CNS: central nervous system
CTLA: Cytotoxic T-lymphocyte antigen
DNAse: deoxyribonuclease
DTR: diphtheria toxin receptor
DTx: diphtheria toxin
EAE: experimental autoimmune encephalomyelitis
FOXP3: Forkhead box p3
IFN: interferon
IFN-γR: interferon-γ receptor
IL: interleukin
i.p.: intra peritoneal
LAG: Lymphocyte-activation gene
LAP: Latency associated peptide: LN, lymph node
MFI: mean fluorescence intensity
MHC: major histocompatibility complex
MNC: mononuclear cells
MOG: myelin oligodendrocyte glycoprotein
MS: Multiple Sclerosis
MT: *mycobacterium tuberculosis*
PBS: phosphate-buffered saline
PD: Program-death
PD-L: Program-death Ligand
p.i.: post-immunization
PLP: proteolipid protein
PMA: phorbol myristate acetate
PTx: pertussis toxin
RR: relapsing-remitting
s.c.: subcutaneous
SC: spinal cord
STAT: Signal Transducers and Activators of Transcription
Th: T helper cells
TGF: Tumor growth factor
Treg: regulatory T
WT: wild type
