# Supplementary material for: Therapeutic role of interferon-γ in experimental autoimmune encephalomyelitis is mediated through a tolerogenic subset of splenic CD11b+ myeloid cells
Source: J Neuroinflammation. 2024 May 31;21:144. doi: 10.1186/s12974-024-03126-3 (PMC11143617; doi:10.1186/s12974-024-03126-3)
Supplement: Supplementary file 1 — Supplementary Material 1 [file 12974_2024_3126_MOESM1_ESM.pdf]

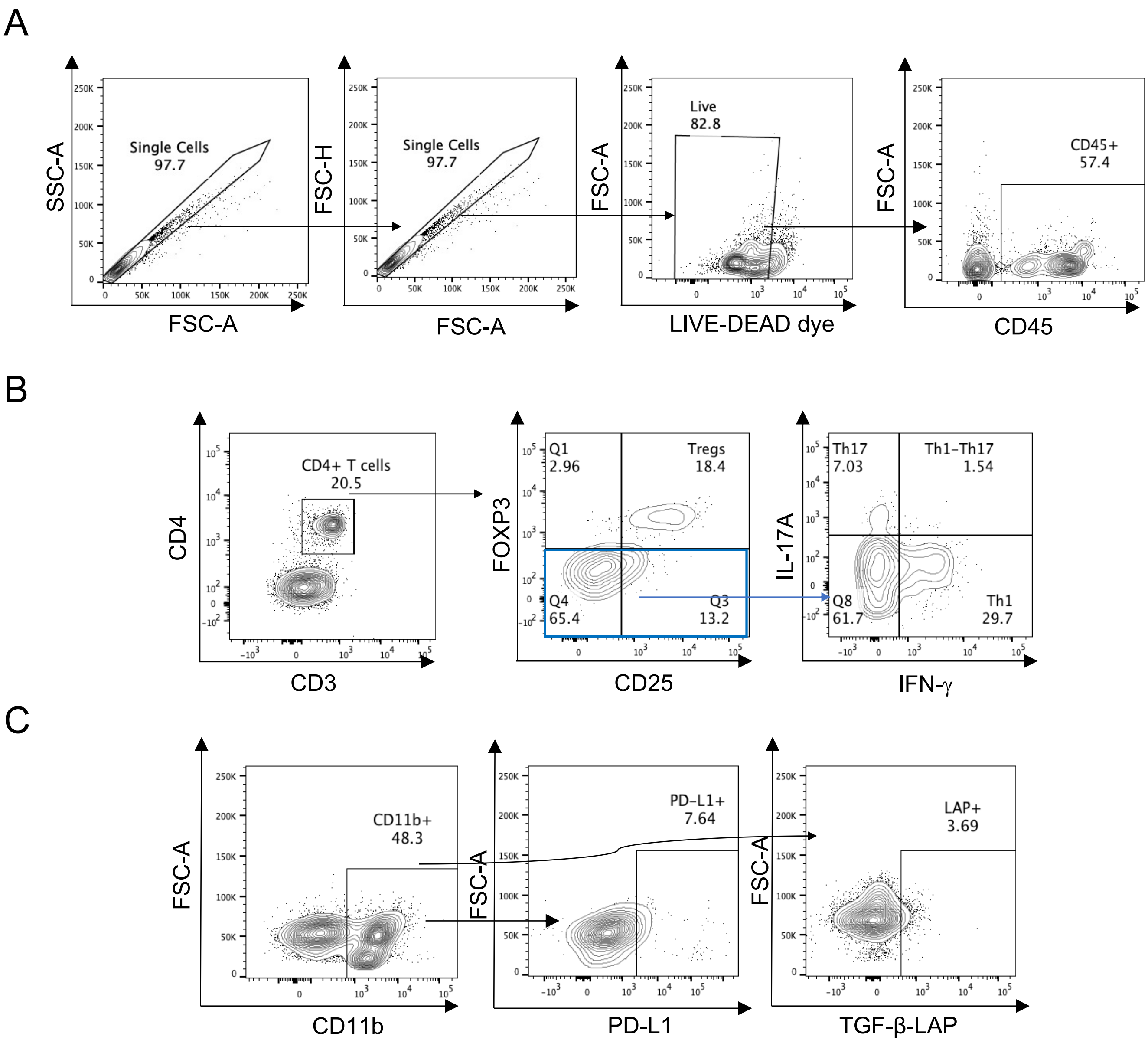

Supplementary Figure 1. Representative flow cytometry analyses strategy for *in vivo* immunophenotyping. (A) General flow cytometry strategy for analysis of live immune cells. **(B)** Gating strategy for $CD4^+$ T cells, Treg cells and effector Th1 and Th17 cells [gated from $FOXP3^-$ (blue box)]. **(C)** Gating strategy for $CD11b^+$ cells expressing PD-L1 or TGF-β-LAP.

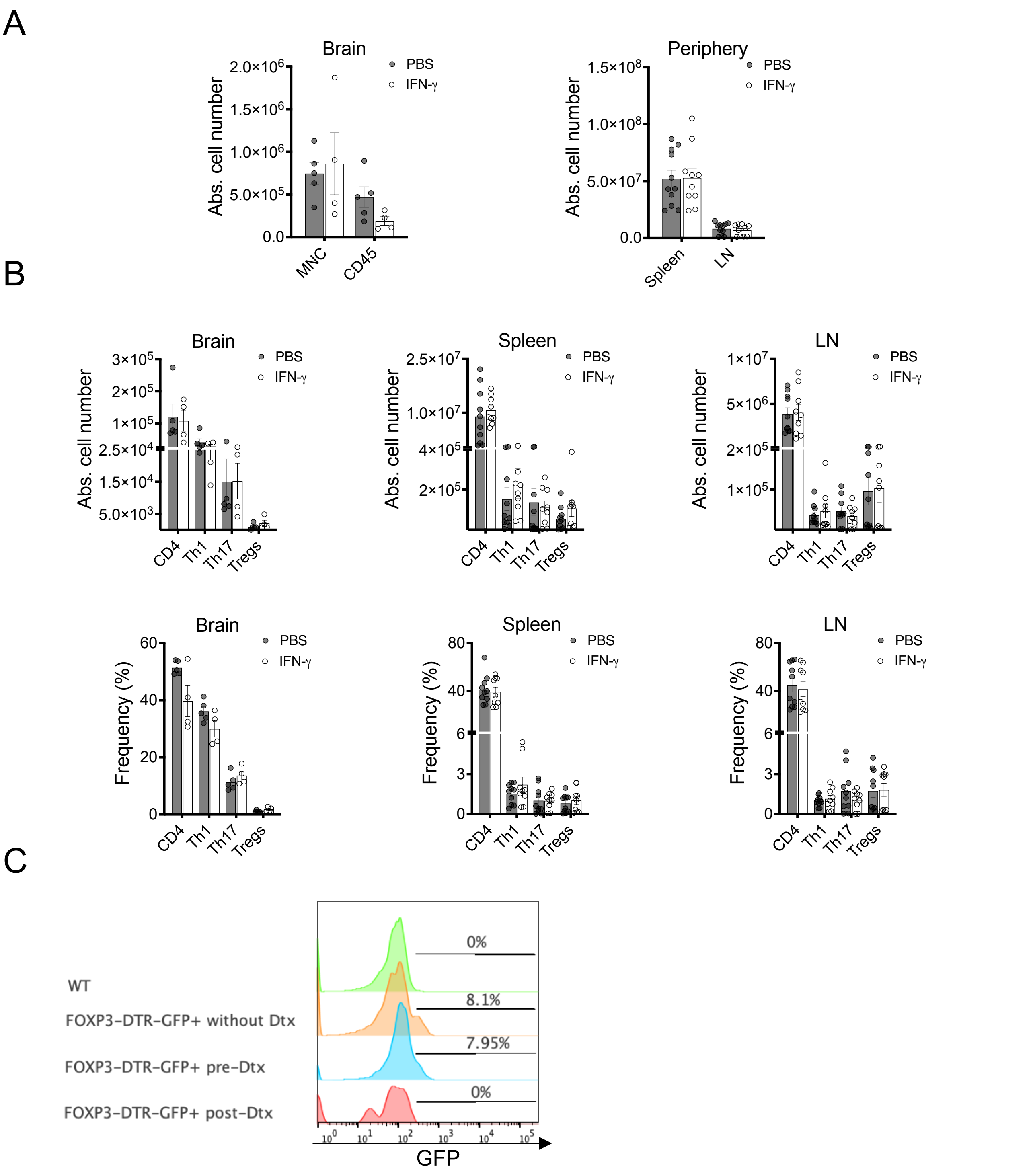

Supplementary Figure 2. Analyses of immune cell populations in CNS and periphery of EAE mice treated with IFN-γ. (A) Absolute number of mononuclear cells (MNC) and CD45$^+$ cells in brain and absolute number of total cells in spleen and lymph nodes (LN) from EAE mice treated with PBS (black bars) or IFN-γ (white bars) determined by flow cytometry. **(B)** Absolute number and frequency of CD4$^+$ T cells, Th1, Th17 and Treg cells in brain, spleen and LN from EAE mice treated with PBS (black bars) or IFN-γ (white bars) determined by flow cytometry. **(C)** Frequency of FOXP3-GFP$^+$ Treg cells determined in blood from WT mice and from FOXP3-DTR mice without diphtheria toxin administration (DTx) and pre and post DTx treatment.

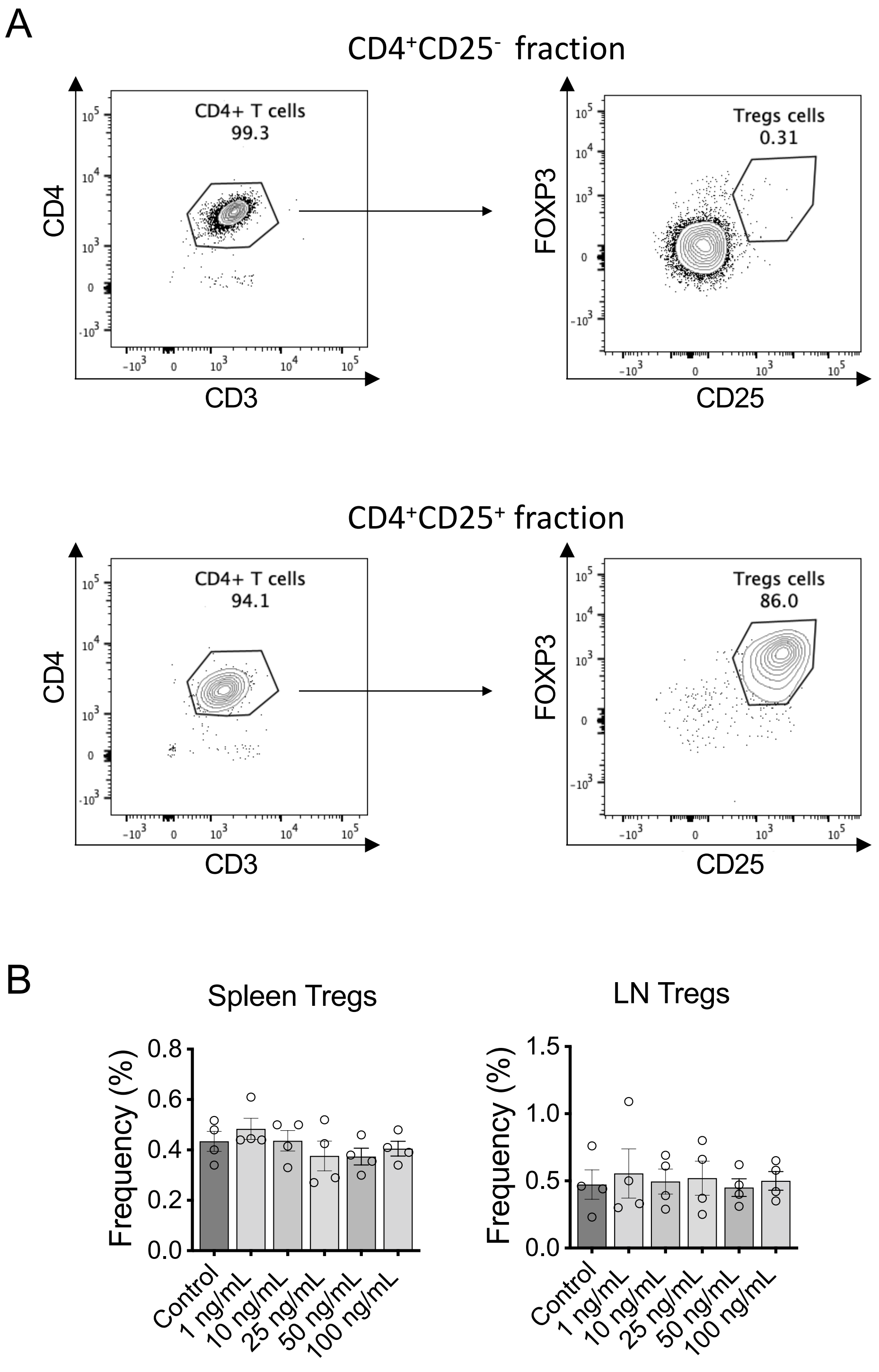

Supplementary Figure 3. Isolation of $CD4^+CD25^-$ and $CD4^+CD25^+$ T cells and Treg cell induction by IFN-γ stimulation. (A) Spleen and draining lymph nodes (LN) were isolated from EAE mice at the peak of disease and used to purify $CD4^+CD25^-$ and $CD4^+CD25^+$ T cell fractions (>90% purity). **(B)** Fraction of $CD4^+CD25^-$ T cells isolated from spleen or LN from EAE mice at the peak of disease were cultured with 2μg/mL of plate-bound anti-CD3 and 1μg/mL of soluble anti-CD28 in the absence (control) or presence of different concentrations (1, 10, 25, 50, 100 ng/mL) of IFN-γ for 3 days. The frequency of Treg cells was determined by flow cytometry.

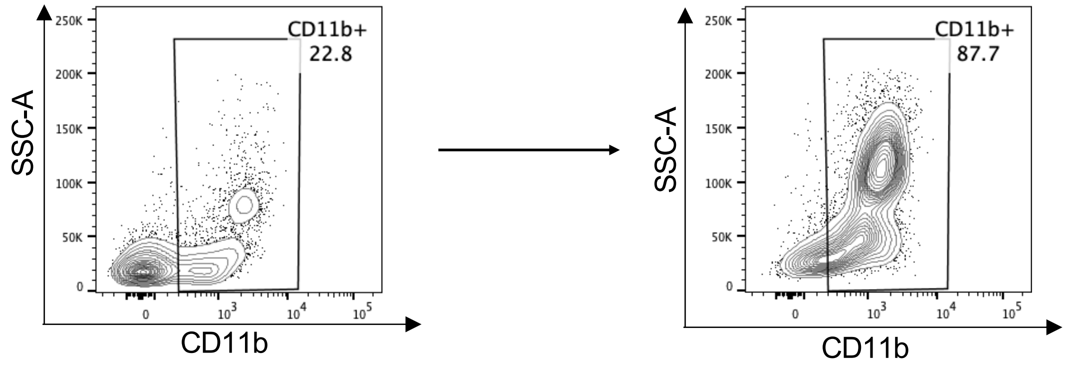

Supplementary Figure 4. Splenic CD11b$^+$ cell purification. CD11b$^+$ cells were isolated from spleen of EAE mice at the peak of the disease by immunomagnetic selection obtaining >87% purity.

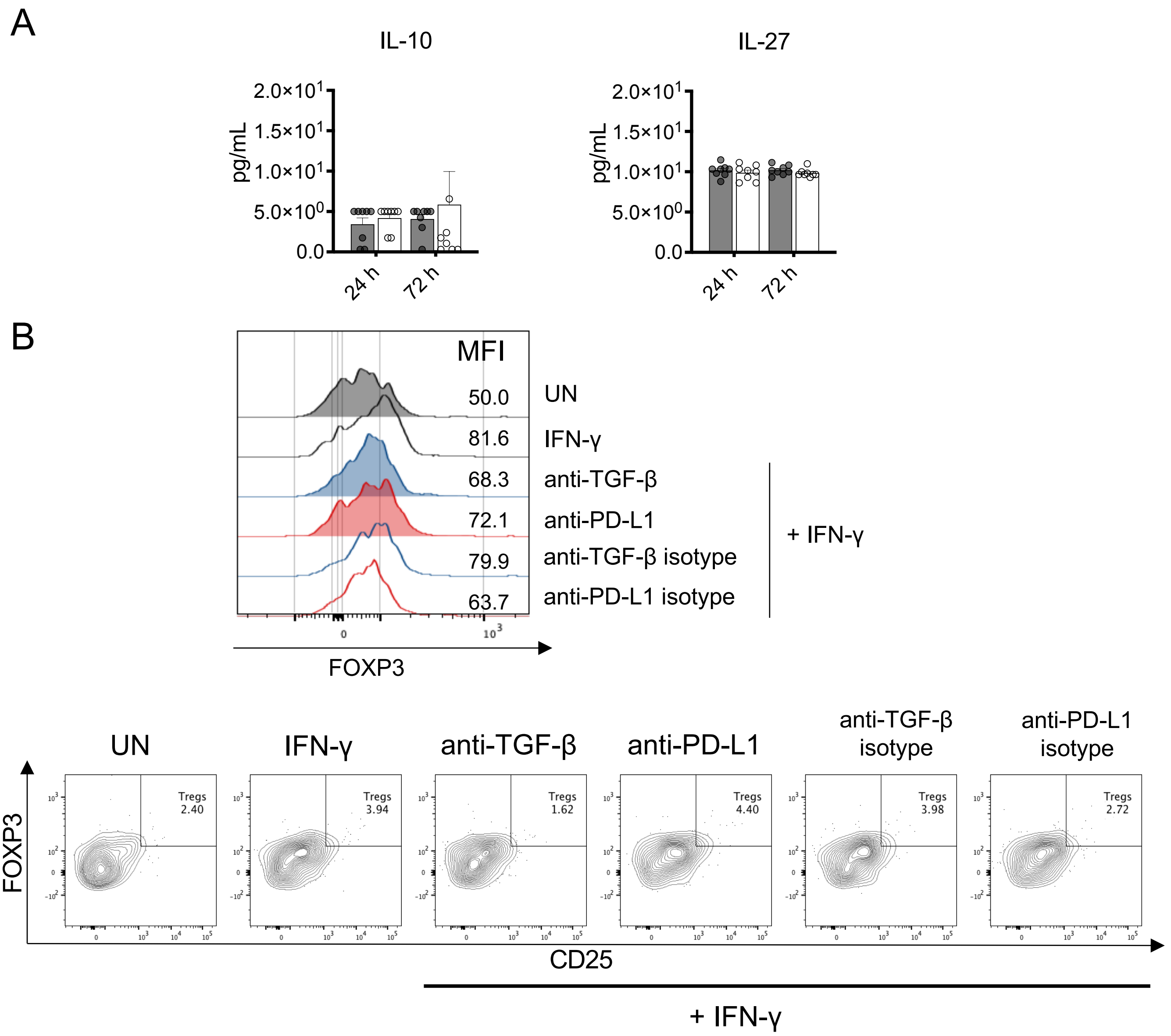

Supplementary Figure 5. Splenic CD11b$^+$ cells from EAE mice preconditioned *in vitro* with IFN-γ induce conversion of CD4$^+$ T naïve cells into Treg cells in a TGF-β-dependent manner. Splenic CD11b$^+$ cells isolated from EAE mice were *ex vivo* stimulated with 25 ng/mL IFN-γ (white bars) or left untreated (black bars) for 24 and 72 hours in the presence of 10 μg/mL $MOG_{33\text{-}55}$. **(A)** Cell culture supernatants were collected at 24 and 72 hours and concentration of IL-10 and IL-27 was measured by multiplex assay (n=8). Splenic CD11b$^+$ cells from EAE mice were preconditioned with 10 μg/mL $MOG_{33\text{-}55}$ and 25 ng/mL IFN-γ for 24 hours, washed, and co-cultured with naïve CD4$^+$ T cells (ratio 1:1) from healthy mice in the presence of 1 μg/mL of soluble anti-CD3 antibody and with either anti-TGF-β antibody (solid blue histogram), anti-PD-L1 antibody (solid red histogram), or corresponding isotype control antibodies (empty blue and red histograms). After 72 hours, the frequency of Treg cells (CD4$^+$CD25$^+$FOXP3$^+$) was determined by flow cytometry. Representative histograms showing mean of fluorescence intensity (MFI) of FOXP3 and frequency of Treg cells is shown.

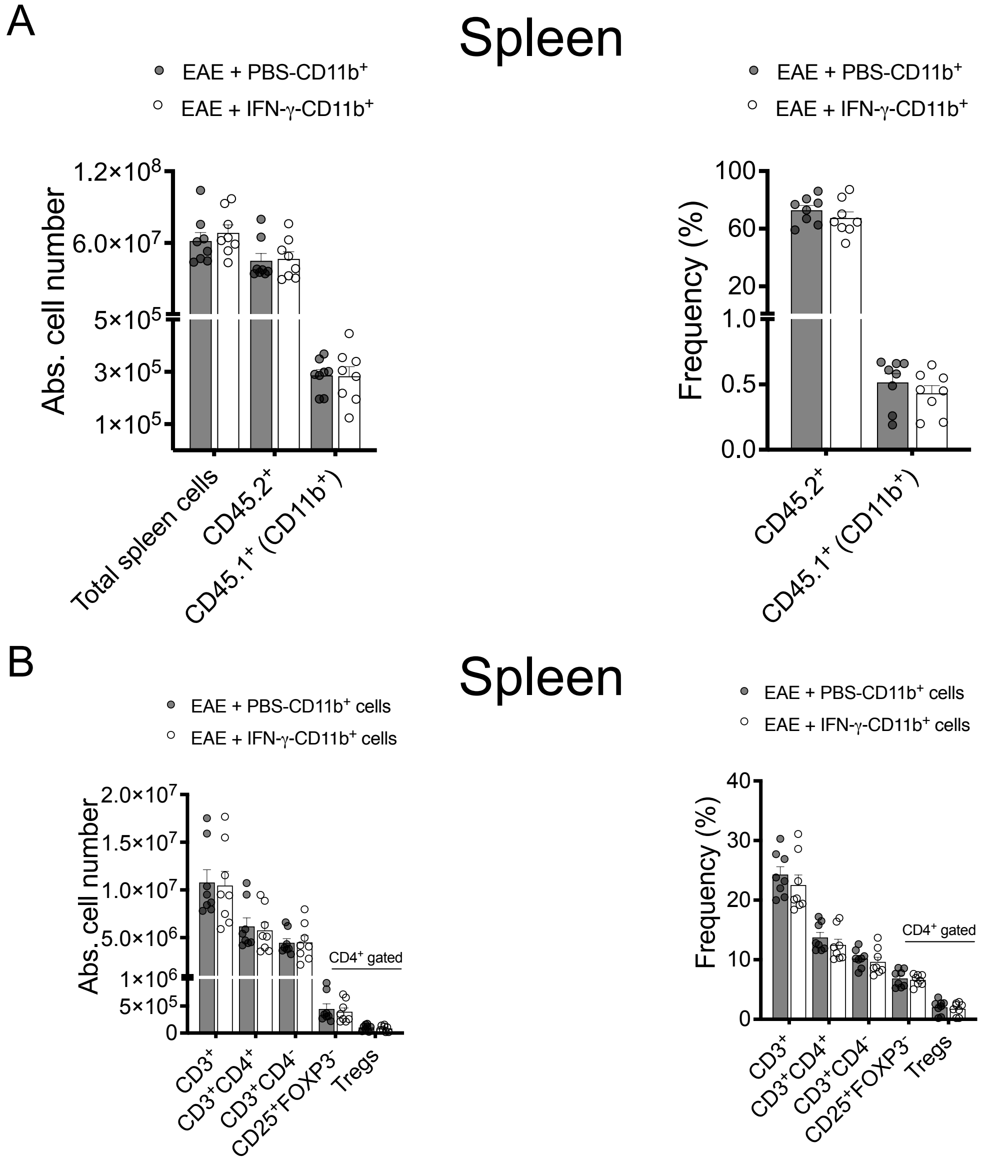

Supplementary Figure 6. Analysis of splenic CD45.2$^+$ cells and CD45.1$^+$CD11b$^+$ cells from EAE recipient mice. Splenic CD11b$^+$ cells (1*10^6) from CD45.1 EAE mice treated for 5 days with IFN-γ or PBS were i.v. transferred into CD45.2 EAE mice at the peak of disease. After three days, splenocytes from recipient EAE mice receiving CD45.1$^+$CD11b$^+$ cell from EAE mice treated with IFN-γ (white bars, n=8) or PBS (black bars, n=8) were isolated and absolute number and frequencies of **(A)** total splenocytes, CD45.2$^+$ cells, and CD45.1$^+$CD11b$^+$ cells, and **(B)** CD3$^+$ T cells, CD3$^+$CD4$^+$ T cells, CD3$^+$CD4$^-$ T cells, CD3$^+$CD4$^+$CD25$^+$FOXP3$^-$ T cells, and Treg cells (CD3$^+$CD4$^+$CD25$^+$FOXP3$^+$), were determined by flow cytometry. Results are shown as the mean ± SEM.
